# Supplementary material for: Treating the disease and meeting the person with the illness-patient perspectives of needs during infective endocarditis, a qualitative study
Source: PLoS One. 2024 Aug 27;19(8):e0309373. doi: 10.1371/journal.pone.0309373 (PMC11349237; doi:10.1371/journal.pone.0309373)
Supplement: Attachment — Submitted filename: Response to Reviewers240312.docx [file pone.0309373.s001.docx]

Response to Reviewers	Halmstad 12th March 2024

*Reveiwer:*

*Based on our evaluation of your manuscript and the information you provided, we do not feel that your submission meets our ethical requirements for human subjects research submissions. PLOS ONE requires that research meets all applicable standards for the ethics of experimentation and research integrity (http://journals.plos.org/plosone/s/human-subjects-research). We reserve the right to reject any submission that does not meet our internal ethical standards, which in some cases are more stringent than local ethical standards.*

*We recognize that you obtained ethical approval from your local ethics committee after you began your study: your ethics approval document is dated 14 Dec 2021, while participants were recruited beginning in Jan 2021.*

Response:

Thank you for bringing the typing error to my attention. The correct version is that Ethical approval was granted 11^th^ January 2022 and the first patient was included 11^th^ February 2022 and the last 23th March 2023..

*Reveiwer:*

*You also state that informed consent was obtained from all participants, which indicates that the participant's data were collected prospectively for research purposes.*

Response:

All patients gave written consent when included in the study. To state the time of inclusion clearly, a table with inclusion date for all participants follows.

| Study patients and the dates of informed consent | |
| --- | --- |
| E1 | 11-02-2022 |
| E2 | 21-02-2022 |
| E3 | 22-02-2022 |
| E4 | 23-02-2022 |
| E5 | 10-03-2022 |
| E6 | 23-05-2022 |
| E7 | 08-06-2022 |
| E8 | 29-07-2022 |
| E9 | 06-09-2022 |
| E10 | 10-01-2023 |
| E11 | 21-11-2022 |
| E12 | 21-11-2022 |
| E14 | 10-01-2023 |
| E15 | 01-02-2023 |
| E16 | 03-02-2023 |
| E17 | 08-02-2023 |
| E18 | 09-02-2023 |
| E19 | 08-03-2023 |
| E20 | 08-03-2023 |
| E21 | 23-03-2023 |

*Reveiwer:*

*For human subjects research, PLOS ONE requires that authors obtain ethics committee approval before beginning the study, and we do not accept retrospective ethical approval.*

Response:

Thank you for bringing the typing error to my attention. The correct version is that Ethical approval was given by the ethical board 11^th^ January 2022 with diary number 2021-06641-01. Inclusion of patients in the study started 11^th^ February 2022.

*Reveiwer:*

*As a result of these concerns, we cannot consider the manuscript for publication at PLOS ONE. I am sorry that we cannot be more positive on this occasion but hope that you understand the reasons for this decision.*

Response:

Thank you for bringing the typing error to my attention. The correct version is that the ethical approval (diary number 2021-06641-01) was given by the ethical board 11^th^ January 2022 Inclusion of patients in the study started 11^th^ February 2022. Previous information was a typing error.

I respect your decision but I wish to correct my case therefore I re-submit my manuscript with the hope of new considerations, based on these responses to Your comments, the attached documentation and the corrections of the manuscript.

Best regards

Helena Lindberg

MD, PhD student Lund university

Senior consultant, Department of Infectious diseases, Region Halland
